# Closely-related *Borrelia burgdorferi* (*sensu stricto*) strains exhibit similar fitness in single infections and asymmetric competition in multiple infections

**DOI:** 10.1186/s13071-016-1964-9

**Published:** 2017-02-06

**Authors:** Evelyn C. Rynkiewicz, Julia Brown, Danielle M. Tufts, Ching-I Huang, Helge Kampen, Stephen J. Bent, Durland Fish, Maria A. Diuk-Wasser

**Affiliations:** 1 Ecology, Evolution, and Environmental Biology Department, Columbia University, 1200 Amsterdam Ave, New York, NY 10027 USA; 2 Yale School of Public Health, 60 College St, New Haven, CT 06510 USA; 3 Friedrich-Loeffler-Institut, Federal Research Institute for Animal Health, Suedufer 10, 17493 Greifswald, Germany; 4 Institute for Molecular Bioscience, University of Queensland, St Lucia, Brisbane, QLD 4072 Australia

**Keywords:** Strain diversity, Co-infection, *Ixodes scapularis*, *Peromyscus leucopus*

## Abstract

**Background:**

Wild hosts are commonly co-infected with complex, genetically diverse, pathogen communities. Competition is expected between genetically or ecologically similar pathogen strains which may influence patterns of coexistence. However, there is little data on how specific strains of these diverse pathogen species interact within the host and how this impacts pathogen persistence in nature. Ticks are the most common disease vector in temperate regions with *Borrelia burgdorferi*, the causative agent of Lyme disease, being the most common vector-borne pathogen in North America. *Borrelia burgdorferi* is a pathogen of high public health concern and there is significant variation in infection phenotype between strains, which influences predictions of pathogen dynamics and spread.

**Methods:**

In a laboratory experiment, we investigated whether two closely-related strains of *B. burgdorferi* (*sensu stricto*) showed similar transmission phenotypes, how the transmission of these strains changed when a host was infected with one strain, re-infected with the same strain, or co-infected with two strains. *Ixodes scapularis*, the black-legged tick, nymphs were used to sequentially infect laboratory-bred *Peromyscus leucopus*, white-footed mice, with one strain only, homologous infection with the same stain, or heterologous infection with both strains. We used the results of this laboratory experiment to simulate long-term persistence and maintenance of each strain in a simple simulation model.

**Results:**

Strain LG734 was more competitive than BL206, showing no difference in transmission between the heterologous infection groups and single-infection controls, while strain BL206 transmission was significantly reduced when strain LG734 infected first. The results of the model show that this asymmetry in competition could lead to extinction of strain BL206 unless there was a tick-to-host transmission advantage to this less competitive strain.

**Conclusions:**

This asymmetric competitive interaction suggests that strain identity and the biotic context of co-infection is important to predict strain dynamics and persistence.

**Electronic supplementary material:**

The online version of this article (doi:10.1186/s13071-016-1964-9) contains supplementary material, which is available to authorized users.

## Background

Wild hosts are often co-infected with multiple parasite species or strains. Within-host interactions between different parasites can have significant implications for disease prevalence and spread [1, 2]. Competitive interactions are expected when pathogens are closely related genetically, have similar infection behavior, share within-host niches, or elicit similar host immune responses [3–5]. However, interactions between co-infecting parasites are often studied in model species that are not their natural hosts [6–8]. Investigating positive and negative interactions between parasites in their natural host is needed to elucidate mechanisms structuring the composition and diversity of pathogen communities in the wild.

Ticks are the most common arthropod vector in temperate climates and *Borrelia burgdorferi* (*sensu lato*), the causative agent of Lyme disease, is the most prevalent vector-borne pathogen in these regions. *Borrelia burgdorferi* (*sensu stricto*) (hereafter, *B. burgdorferi*) is the sole causative agent of Lyme disease in North America [9]. In the eastern United States, *B. burgdorferi* is vectored by the generalist tick *Ixodes scapularis*, which parasitizes a wide variety of vertebrate hosts at each life stage [10, 11]. *Peromyscus leucopus*, the white-footed mouse, is an integral part of the life-cycle of both *I. scapularis* and *B. burgdorferi*. These mice are the primary hosts of larval and nymphal tick stages, are highly competent hosts for the amplification of *B. burgdorferi* strains, and are abundant in the northeastern United States [11–13]. In addition, *P. leucopus* does not develop arthritic pathology, as seen in many laboratory mouse strains [14], suggesting significant differences in the physiological response to *B. burgdorferi* infection.

*Borrelia burgdorferi* shows high variability in genes specific to its ability to infect the tick vector, the vertebrate host, and evading the host immune response. The *ospC* locus of *B. burgdorferi* has been of special interest in studies of spirochetal diversity due to the important role it plays in mediating the interaction between distinct strains in mixed infections via the host immune response [15–17]. At least 22 major *ospC* groups have been described with 15 common in the northeastern United States [16, 18]. *ospC* is expressed during tick feeding and during the first 10 days of infection and is essential for transmission of the spirochete from tick-to-host [19]. The protein is a major antigenic target for the vertebrate immune system and strains with particular *ospC* genotypes (e.g. A, B, I and K) have been associated with invasiveness and disseminating infections in humans [20, 21].

Possibly due to the generalist nature of its vector and the resulting wide range of zoonotic hosts, most strains of *B. burgdorferi* are also generalists, although the frequency of these strains does vary across different reservoir host species [12, 16]. Interactions among *B. burgdorferi* strains within the host, tick, or during transmission may partially account for the maintenance of this diversity. Derdakova et al. [22] demonstrated that *B. burgdorferi* strains belonging to two distantly related genotypes had very different transmission phenotypes and that co-infection reduced transmission of both strains. Comparatively, competition among closely related genotypes is expected to be stronger as they are more likely to share similar resources and more cross-reactivity in strain-specific antibodies [15].

Using a laboratory system including the natural vector and host species for *B. burgdorferi*, we examined the transmission dynamics of single infections as well as homologous and heterologous sequential infections of two closely related strains of *B. burgdorferi*. Our goals were to (i) analyze differences in transmission phenotype between two closely-related genotypes, (ii) characterize differences in host-to-tick transmission when hosts were infected once with one strain, re-infected with the same strain, or co-infected with two strains, and (iii) use a simple simulation model to expand on the results of the laboratory study to examine the expected patterns of long-term persistence of each strain based on the observed interactions in the laboratory.

## Methods

### *Borrelia burgdorferi* strains

Two isolates of *B. burgdorferi* were used in the study, BL206 and LG734. These strains are classified as RST Type I, in reference to restriction fragment-length polymorphism of the 16S-23S ribosomal DNA intergenic spacer, and form a monophyletic group with five additional strains [23]. BL206 represents the 16S-23S rDNA intergenic spacer genotype 1 (IGS1) and *ospC* group A [17, 24, 25], while LG734 was genotyped as IGS3 and *ospC* group B. Strain BL206 is a clinical isolate obtained from the blood of a Lyme disease patient at Westchester Medical Center in Valhalla, NY, and cultured in BSK II media [26]. Initially, nymphs infected with BL206 were obtained by allowing larvae to feed on C3H/HeNCr1 mice inoculated intraperitoneally with the BL206 isolate [22, 27]. At the time of this experiment, BL206 had been passaged six times using infected *I. scapularis* ticks in C3H/HeNCr1 mice. Strain LG734 was isolated from a wild *I. scapularis* nymph collected from Lake Gaillard in North Branford, CT, USA during the summer of 2007. A single wild-collected nymph was used to infect a CB-17/SCID mouse. Larvae fed on this mouse were used for this experiment after molting. *Peromyscus leucopus* mice were obtained from the *Peromyscus* Genetic Stock Center (Columbia, SC, USA). While changes in plasmid content or other virulence factors could have occurred in culture or *Mus musculus* mice [28, 29], both strains successfully transmit between *P. leucopus* and ticks, so we expect our observations represent natural interactions of two disseminating strains. PCR analysis of nymphs used to infect mice in this study showed infection prevalence was 95% or higher for both strains, giving us high certainty of tick-to-mouse infection.

To maintain ticks for laboratory infections, field-collected adult *I. scapularis* were collected from the field and fed on uninfected New Zealand white rabbits. Larvae hatched from these egg clutches were confirmed to be uninfected with any pathogens and are then fed on mice. Ticks are therefore outbred which maintains the genetic diversity found in natural *I. scapularis* populations.

### Host study species

Adult male and female specific-pathogen-free *P. leucopus* (LL stock) mice were obtained from the *Peromyscus* Genetic Stock Center and bred at Yale University. All mice were handled humanely in accordance with the Yale University Institutional Animal Care and Use Committee guidelines.

### Experimental design

**Fig. 1:**
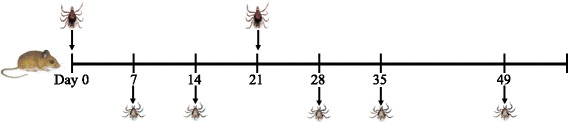
Illustration of laboratory infection of hosts and xenodiagnoses. Infected nymphs were used to infect hosts at days 0 and 21 (uninfected nymphs were used on day 21 for single-infection controls). Uninfected larvae were used to assess pathogen transmission at days 7, 14, 28, 35 and 49

Six treatment groups were established with four *P. leucopus* in each. Infections were established via feeding infected nymphal ticks on each mouse (12 nymphs per mouse; Fig. 1). Nymphs were allowed to feed to repletion and then collected; for full description of tick collection methods see [22]. Single-infection controls received nymphs infected with either strain BL206 or LG734 at day 0 and subsequently received clean nymphs at day 21. Homologous infection groups received nymphs infected with the same strain at day 0 and 21, while heterologous groups received nymphs of the different strain at day 21. The transmissibility of the strains was assessed using xenodiagnosis by placing 100 clean larvae on each mouse on days 7, 14, 28, 35 and 49 after the initial infection. Larvae were allowed to feed to repletion, engorged larvae were collected daily and allowed to molt into nymphs (Fig. 1). Random samples of 20 molted nymphs per mouse were individually tested by multiplex PCR for the presence of both strains of *B. burgdorferi*.

### DNA extraction

DNA was extracted from individual ticks using the DNeasy Blood and Tissue Kit (Qiagen, Valencia, CA, USA) using a slightly modified manufacturer’s protocol [22, 30]. Each tick was initially frozen in liquid nitrogen and ground in a 1.5 ml centrifuge tube using a plastic pestle. Proteins were degraded overnight at 56 °C in 180 μl of ATL buffer (Qiagen) and 40 μl of proteinase K (Roche Applied Science, Indianapolis, IN, USA); the remainder of the extraction followed the manufacturer’s protocol. DNA from each tick was diluted in 50 μl elution buffer (Qiagen) and stored at 4 °C.

### PCR amplification

*Borrelia burgdorferi* strains BL206 and LG734 were detected in a nested-multiplex PCR protocol amplifying a region of the *ospC* locus. This sensitive method allowed for simultaneous individual detection of both strains, including mixed infections. The first round of PCR followed conditions described previously [17]. This first round of PCR used primers ospC-F (5′-ATG AAA AAG AAT ACA TTA AGT GC-3′ corresponding to positions 306–328 of U01894) and ospC-R (5′-ATT AAT CTT ATA ATA TTG ATT TTA ATT AAG G-3′ corresponding to positions 963–933 of U01894). Samples were amplified using HotStarTaq (Qiagen) under the following conditions: an initial denaturation step of 95 °C for 15 min followed by 10 cycles of 94 °C for 30 s, 52 °C for 30 s, 72 °C for 1 min; 30 cycles of 94 °C for 30 s, 50 °C for 30 s, 72 °C for 1 min; and a final elongation step of 72 °C for 5 min.

The second step of the nested PCR protocol was a multiplex PCR using two forward primers (OspC-A237F: 5′-GAT ACC GAA AAT AAT CAC AAT GGA-3′ and OspC-B500F: 5′-AAA GTT GTC CGG ATC ATT AGA AAG-3′) and a single reverse primer (OspC-Rn: 5′- TTG ATT TTA ATT AAG GTT TTT TTG G-3′) corresponding to positions 948–924 of U01894 [23]. These primers were specifically designed to distinguish between the two *B. burgdorferi* strains, BL206 and LG734, belonging to *ospC* types A and B, respectively. OspC-A237 amplified a product of 382 bp signifying infection with strain BL206, while OspC-B500 amplified a product of 122 bp signifying infection with strain LG734. Samples were amplified using ImmoMix™ Red (Bioline, London, UK) under the following conditions: an initial denaturation step of 95 °C for 15 min followed by 40 cycles of 94 °C for 30 s, 52 °C for 30 s, 72 °C for 1 min and a final elongation step of 72 °C for 5 min. All PCR products were subjected to gel electrophoresis and infection status was determined by single or multiple bands at the aforementioned lengths. Three positive controls, BL206 only, LG738 only, and mixed strains, and one negative control, sterile water, were included in each PCR reaction to confirm success of the PCR and to determine band size(s) in each sample.

### Statistical analysis

To assess whether co-infection with the homologous or heterologous strain influenced the total transmission of each *B. burgdorferi* strain to feeding ticks, we compared the proportion of all ticks infected with each strain (the sum of singly-infected ticks from a strain and co-infected ticks, divided by the total number of ticks from the host; Additional file 1) with the respective single-infection controls. We used Generalized Estimating Equations (GEE; binomial variance distribution, Wald Chi-squared test to determine significance) using the *geepack* package [31] in R [32]. This approach analyzes clusters of correlated data, such as longitudinal measures taken from the same individuals over time, without the need to define an error structure, only the means at each sampling time. An independent correlation matrix was specified in the model to remove constraints on the analysis of a specific correlation among repeated samples; this model had the lowest QIC (Quasi-Akaike Information Criterion) compared to models with other correlation structures, indicating the best GEE model fit to the data. We utilised the same statistical method to compare transmission phenotypes between the two single-infection controls.

To assess whether the interaction between strains was manifested during transmission to feeding ticks, we used a Fisher’s exact test to compare the observed prevalence of co-infected ticks to the expected prevalence if both strains were transmitted independently using the *exact2×2* package in R [33] (minimum likelihood method to determine significance, odds ratio test statistic). Expected co-infection prevalence was calculated as the product of the proportion of ticks singly-infected with each strain from each individual mouse, multiplied by the total ticks from each group at each of the three time points post-secondary infection.

### Simulation of strain maintenance

**Fig. 2:**
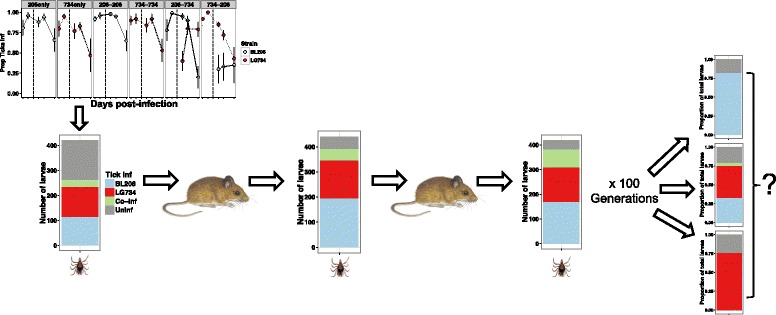
Illustration of the simulation model. Each generation consisted of 100 mice. Nymphs from the previous generation were used to create a virtual pool from which nymphs were drawn to infect mice at the next generation (with first generation using results from the final day of the laboratory experiment). One nymph was randomly drawn at day 0 and again at day 21 for the infections. Possible outcomes include extinction of either strain or coexistence

To investigate the effect of co-infection on the long-term coexistence of these two closely-related strains, we performed a numerical simulation to repeat the experiment described here for 100 generations using the virtual pool of ticks from the previous generation as the source for infection in the next generation (Fig. 2). In each generation, we simulated 100 uninfected mice and sequentially put two nymphs on each mouse at day 0 and day 21. The nymphs were randomly chosen from a virtual pool of nymphs with the same infection distribution as in the final day of the previous generation (day 49), with the first simulated generation using the results from the laboratory study. As in the laboratory experiment, there were four types of infected nymphs possible in the simulation: uninfected, infected with BL206 only, LG734 only, and co-infected. Seven types of mouse infections could be produced based on order of infections (uninfected, BL206 only, LG734 only, BL206-BL206, LG734-LG734, BL206-LG734, or LG734-BL206). At days 7, 14, 28, 35 and 49 of the simulation, we simulated xenodiagnoses by infesting each mouse with 20 uninfected larvae that acquired the pathogen infection following the trajectories in the observed experiment. Stochasticity was introduced into the model in each generation by choosing 200 out of 10,000 ticks produced in each previous generation to infect the 100 mice in each subsequent generation.

Because the experimental methods could not measure transmission via co-infected nymphs, we assume in the model that only one strain will be transmitted in each tick-to-host transmission event if a co-infected tick was randomly chosen from the pool, i.e. a co-infected tick does not pass on both pathogens to the host. We introduced a parameter *p* to denote the probability of a mouse becoming infected with strain LG734 from a co-infected nymph; 1-*p* denoted the probability of infection with strain BL206. We varied *p* between 0 and 1 to explore if certain values would enable coexistence of the two strains or extinction of one strain. We performed the simulation over 10,000 ensembles to extract the global trend out of the stochasticity. The mean prevalence of each tick and mouse infection type from the 10,000 repeated simulations is presented. For full model code please see Additional file 2.

## Results

**Fig. 3:**
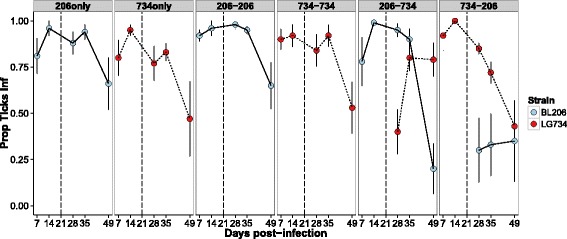
Transmission dynamics of strains BL206 and LG734 over the course of the laboratory experiment for each infection group (*panels*). The total proportion of ticks infected with strain BL206 is shown in *blue* (*solid line*) and LG734 in *red* (*dashed line*). The proportion of ticks infected with each strain is the sum of those singly-infected with a strain and co-infected ticks

In the laboratory, mice acquired infections of both strains BL206 and LG734 and transmitted them at high rates, with infection prevalence in xenodiagnostic ticks being above 75% for most individuals at the first sampling day post-infection (day 7; Fig. 3). For both strains, the single-infection controls showed high transmission intensity from days 7 to 35 and then a decrease at day 49.

### Strain dynamics

The two single-infection controls showed similar transmission phenotypes. There was no significant difference in the proportion of ticks infected over the course of the experiment between single-infection controls for strains BL206 and LG734 (Table 1, 'Controls'). For both strains, there were no significant differences between the homologous transmission groups and the single-infection controls. In heterologous infections, neither strain differed from the single-infection control if they infected first. For the strains that infected second, strain BL206 transmission was significantly lower than the single-infection control (Wald = 22.89, *P* < 0.0001; Table 1 *Post-hoc*, Fig. 3), but there was no difference in transmission for strain LG734 compared to the single-infection control for that strain.

**Table 1:** Results of the Generalized Estimating Equation (GEE) analyses of transmission dynamics between the two single infection controls and between each genotype in mixed infections and the respective single-infection control (Overall). *Post-hoc* analyses compared transmission in *each* mixed infection an the respective single-infection control

	Overall		*Post-hoc*		
Strain	Wald *χ* ^2^	*P-*value	Group	Wald *χ* ^2^	*P*-value
Controls	1.52	0.22			
BL206	31.3	< 0.0001	734–734	0.6	0.44
			206–734	1.22	0.27
			734–206	22.89	< 0.0001
LG734	4.42	0.22	206–206	0.9	0.342
			206–734	1.29	0.257
			734–206	0.05	0.823

### Observed *vs* expected co-infection

**Fig. 4:**
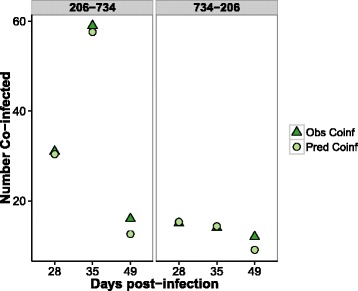
The observed and expected number of ticks co-infected from each heterologous infection group at the three time points post-secondary infection (observed values: *dark green triangles*, expected values: *light green circles*)

The observed co-infection prevalence in ticks in the heterologous infection groups were not significantly different from the expected co-infection prevalence in either heterologous infection group (Table 2, Fig. 4).

**Table 2:** Observed and expected numbers of co-infected ticks from each heterologous infection group at each time point post-secondary infection. Results of Fisher’s exact test comparing expected and observed co-infection prevalence given (odds ratio, *P*-value)

Infection treatment	Days post-infection	Obs. ticks co-infected	Expected ticks co-infected	Odds ratio	*P-*value
206–734	28	31	30.4	0.949	1
	35	59	57.6	0.939	1
	49	16	12.6	0.777	0.7
734–206	28	15	15.3	1	1
	35	14	14.3	1	1
	49	12	9.1	0.708	0.6

### Simulation of strain persistence over time

**Fig. 5:**
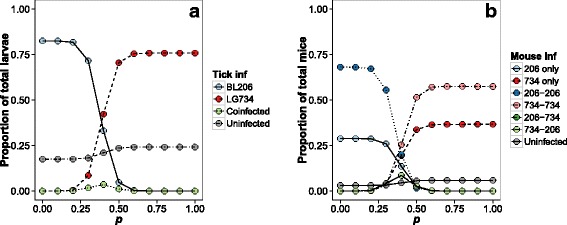
Results of the simulation model in response to variation in *p*, the probability of a mouse becoming infected with strain LG734 from a co-infected nymph (1-*p* denotes probability of infection with strain BL206). Infection prevalence of each type of infection in ticks (**a**), and each type of infection in host determined by all possible sequential infections (**b**)

The simulation model showed that strain BL206 would become extinct from the population unless BL206 had higher probability of tick-to-host transmission than strain LG734 (Fig. 5a). By changing the value of *p*, the probability of LG734 being transmitted from a co-infected tick, we found that both strains were predicted to coexist when *p* was between 0.2 and 0.6 when considering model stochasticity. This indicates that a selective advantage in BL206 tick-to-host transmission would be required to counteract the host-to-tick advantage of LG734 and allow both strains to coexist. Because the probabilities of a mouse becoming infected with either strain first or second were independent, the frequencies of co-infected mice infected with BL206 first or LG734 first were almost identical (Fig. 5b).

## Discussion

Using a natural host-vector-pathogen system we demonstrated that two closely related, disseminating strains of *B. burgdorferi* show similar transmission phenotypes when natural reservoir hosts are singly-infected or infected twice with the same strain. However, strain LG734 displayed a competitive advantage over strain BL206 when LG734 infected the host first. The results of a simple simulation model suggest that this interaction would lead to the extinction of strain BL206 unless there was a tick-to-host transmission advantage to strain BL206. These results have implications for predictions of disease dynamics and maintenance of diversity in mixed-strain pathogen populations.

Strains BL206 and LG734 showed independent infection dynamics when hosts were infected multiple times with the same strain or co-infected with both strains, except when LG734 infected first and BL206 second. In this case, the total number of ticks infected with strain BL206 was always lower than for strain LG734. However, we found no significant differences between observed and expected co-infection prevalence in nymphs, indicating independent transmission of each strain from host-to-tick. Previous analyses of multiple strain infections of *B. afzelii* in wild hosts showed more closely-related strains were less likely to be found together [15]. The authors hypothesized that host antibody cross-reactivity between closely-related strains may be responsible for this pattern. Our results support the hypothesis that closely-related strains would be subject to within-host competition, as strain BL206 had much lower transmission when infecting after strain LG734. However, it is unclear the mechanism by which the same pattern of suppressed transmission was not observed in LG734 when BL206 was the first to infect. Future investigation into antibody similarity and cross-reactivity between these two strains or differences in innate immune response to each strain will be needed to address this asymmetric competition.

The simulation model showed that the transmission dynamics described in the laboratory experiment simulated for 100 generations would lead to extinction of strain BL206 and dominance of strain LG734. In our simulation, only a tick-to-host transmission advantage to strain BL206, the less-competitive strain in host-to-tick transmission, would lead to maintenance of strain BL206 in the population and allow for coexistence between the two strains. This could exemplify a pathogen life-history trade-off between tick-to-host and host-to-tick transmission, postulated by Tonetti et al. [34] as a mechanism for maintenance of strain diversity.

Other possible mechanisms not tested here would also allow for maintenance of multiple strains, including negative frequency dependent selection, multiple niche polymorphism, or variation in phenology of tick life-stages. The first mechanism describes multiple strains being maintained through time by rare advantage, where rare strains become more common when the host population is adapted or primed to respond to common strains [35, 36]. The second mechanism proposes that *B. burgdorferi* diversity can be maintained by variation in a host’s ability to clear certain pathogen strains along with variation in pathogen strain ability to infect multiple host species [16, 37]. Lastly, synchronous feeding activity of larvae and nymphs can facilitate the maintenance of less competitive or rapidly-cleared strains by allowing them to be transmitted to larvae feeding on the same host [38–40]. Opportunities for co-feeding, where ticks feeding closely together transfer pathogens through a localized infection, are also enhanced when larvae and nymphs feed synchronously [41, 42]. Disentangling the multiple mechanisms likely responsible for the maintenance of *B. burgdorferi* diversity will require more extensive laboratory experiments like the one presented here and more complex modeling frameworks. Additional host species should also be studied to assess the role of strain-specific variation in host susceptibility, persistence and transmission to ticks for the maintenance of *B. burgdorferi* diversity.

Simultaneous co-infections were not included in this study but would be an interesting condition to include in future studies. With the current data we cannot predict if strain LG734 would show a transmission advantage over BL206, as seen when LG734 infected first, or if their transmission dynamics would be independent, as was the case when BL206 infected first. A laboratory study of two unrelated tick-borne pathogens, *B. burgdorferi* and *Babesia microti*, showed transmission facilitation of *B. microti* when a host was simultaneously co-infected with both pathogens compared to single infections [1]. Considering co-infection with two strains of the same pathogens, antibody profiles could differ between simultaneous and sequential infections, leading to different cross-reactivity and impacts on strain infection or transmission success [15]. We also only analyzed pathogen transmission to ticks, but additional investigation into tissue tropism of each strain within the host, such as in blood, skin, and joints, could elucidate mechanism of niche differentiation or direct competition among host tissues. Future inclusion of simultaneous co-infection and analysis of pathogen load in various tissues would allow more detailed investigation of infection dynamics and behavior of these two strains.

An advantage of our study design was the use of the natural host and vector to investigate pathogen strain dynamics producing results that more closely mimic natural conditions than studies using laboratory strains of *Mus musculus* or syringe injections. Parallel infections of *B. burgdorferi* in *P. leucopus* and a common *M. musculus* strain found very different transmission dynamics in the natural *vs* laboratory host [25]. Previous studies have also demonstrated that tick saliva has widespread effects on the host immune response [43–45] and unnatural transmission of *B. burgdorferi* (e.g. injections or skin grafts) can lead to substantial differences in infection outcomes [46]. For example, Devevey et al. [6] found that when C3H/HeJ *M. musculus* were injected with different strains of *B. burgdorferi*, the strain that infected first was the strain that best and often singly-infected multiple tissues within the host. In contrast, here, strain LG734 suppressed transmission of BL206 when it infected first, however, LG734 was not suppressed when BL206 infected first. These results illustrate strain-specific differences in competitive ability rather than complete advantage to the primary infecting strain, and is consistent with the high levels of co-infection observed in nature [47].

## Conclusions

In conclusion, two closely related strains of *B. burgdorferi* produced nearly identical infection phenotypes when infecting a host alone, however strain LG734 suppressed strain BL206 when LG734 infected first. This suggests that strain identity and the biotic context of co-infection is important to predicting strain dynamics. Additional transmission experiments with other strains of this and other pathogens in *P. leucopus* and other natural hosts should be performed using vector infection to fully understand how multiple pathogen strains interact and the mechanisms involved in the maintenance of a diverse strain community.

## Additional files

Additional file 1: Data from laboratory infections used in statistical analyses. (CSV 4 kb)
Additional file 2: Model code for simulation of strain persistence. (R 3 kb)
